# The Tablet-Based, Engagement, Assessment, Support, and Sign-Posting (EASSi) Tool for Facilitating and Structuring Sexual Well-Being Conversations in Routine Prostate Cancer Care: Mixed-Methods Study

**DOI:** 10.2196/20137

**Published:** 2020-12-04

**Authors:** Eilís McCaughan, Carrie Flannagan, Kader Parahoo, John Connaghan, Roma Maguire, Mary Steele, Samantha Thompson, Suneil Jain, Michael Kirby, Nuala Brady, Seán R O'Connor

**Affiliations:** 1 Institute of Nursing & Health Research Ulster University Newtownabbey United Kingdom; 2 Department of Computing and Information Sciences University of Strathclyde Glasgow United Kingdom; 3 Faculty of Social and Human Sciences Centre for Clinical and Community Applications of Health Psychology University of Southampton Southampton United Kingdom; 4 Urology Department Belfast City Hospital Belfast United Kingdom; 5 Centre for Cancer Research and Cell Biology School of Medicine, Dentistry and Biomedical Sciences Queen's University Belfast Belfast United Kingdom; 6 Clinical Oncology Northern Ireland Cancer Centre Belfast City Hospital Belfast United Kingdom; 7 Faculty of Health and Human Sciences University of Hertfordshire Hatfield United Kingdom; 8 The Prostate Centre London United Kingdom; 9 Northern Heath and Social Care Trust Antrim United Kingdom; 10 Centre for Public Health Royal Victoria Hospital Queen's University Belfast Belfast United Kingdom

**Keywords:** prostate cancer, sexual well-being, quality of life, communication

## Abstract

**Background:**

Long-term side-effects associated with different prostate cancer treatment approaches are common. Sexual challenges are the most frequently occurring issues and can result in increased psychological morbidity. It is recognized that barriers to communication can make initiating discussions around sexual concerns in routine practice difficult. Health care professionals need to routinely initiate conversations, effectively engage with patients, and assess needs in order to provide essential support. One proposed method that could support health care professionals to do this involves the use of prompts or structured frameworks to guide conversations.

**Objective:**

This study aimed to assess feasibility, acceptability, and satisfaction with the tablet-based Engagement, Assessment, Support, and Sign-posting (EASSi) tool designed to facilitate and structure sexual well-being discussions in routine prostate cancer care.

**Methods:**

Health care professionals (n=8) used the EASSi tool during 89 posttreatment appointments. Quantitative data were recorded based on program usage and surveys completed by health care professionals and patients. Qualitative data exploring perceptions on use of the tool were gathered using semistructured interviews with all health care professionals (n=8) and a sample of patients (n=10).

**Results:**

Surveys were completed by health care professionals immediately following each appointment (n=89, 100%). Postal surveys were returned by 59 patients (66%). Health care professionals and patients reported that the tool helped facilitate discussions (81/89, 91% and 50/59, 85%, respectively) and that information provided was relevant (82/89, 92% and 50/59, 85%, respectively). The mean conversation duration was 6.01 minutes (SD 2.91). Qualitative synthesis identified the tool’s ability to initiate and structure discussions, improve the “depth” of conversations, and normalize sexual concerns.

**Conclusions:**

The EASSi tool was appropriate and acceptable for use in practice and provided a flexible approach to facilitate routine brief conversations and deliver essential sexual well-being support. Further work will be conducted to evaluate the effectiveness of using the tablet-based tool in prostate cancer care settings.

## Introduction

### Background

Prostate cancer is the single most common cancer among men [1,2], and long-term side-effects associated with different treatment approaches are common [3]. Sexual challenges are the most frequently occurring sequelae [4,5], with rates of sexual dysfunction having a moderate to severe impact on quality of life of 31%-64% reported after radical prostatectomy and external beam radiotherapy [6,7]. In a recent large-scale survey, 81% of men reported poor sexual function after treatment [8]. Changes to sexual function are subsequently regarded as a major issue that can result in higher levels of anxiety, depression, relational dissatisfaction, and reduced overall quality of life [9,10]. Current guidelines [11,12] support delivery of psychosexual care for prostate cancer patients and recommend a minimal level of support throughout all phases of care. This includes provision of information tailored to needs, advice about potential adverse effects of treatment, and ongoing access to specialist services including erectile dysfunction clinics. Despite this, sexual aspects of recovery are often not discussed [13-15], and services are not provided consistently across settings. Men frequently report that they do not receive adequate information and support to manage sexual concerns. This has been associated with increased psychological morbidity [16,17].

It is recognized that initiating discussions around sexual concerns in routine practice can be problematic [18-20]. Health care professionals can regard patients’ sexual lives as being too personal to ask about [21,22] and may feel unequipped to deal with sexual issues, reporting a lack of resources to offer patients if they identify a problem [23]. There is evidence that attitudinal barriers and beliefs can lead health care professionals to actively avoid initiating discussions [24]. Fear of personal embarrassment or fear of causing offence and uncertainty over whose role it is to discuss sexual issues have been identified as possible reasons for the low profile of sexual concerns [20]. Men can also feel uncertain about discussing concerns and may not be fully aware of the potential side-effects of treatment on sexual function. Despite these barriers, given their frequency and substantial impact [9], sexual concerns should be discussed with all patients. To adequately address sexual well-being issues, health care professionals need to initiate conversations and effectively engage with patients and assess needs in order to provide essential support and appropriate evidence-based management [25]. One proposed method that could support health care professionals to do this is the use of prompts or structured frameworks to guide conversations [26,27]. This approach may enhance patient-provider communication, particularly around complex or sensitive sexual issues by ensuring a more standardized provision of information [28].

### Objectives

The systematically developed online Engagement, Assessment, Support, and Sign-posting (EASSi) tool was designed to facilitate and structure brief sexual well-being discussions in routine prostate cancer care. An iterative and theory-based process modeled on the person-based approach was used to inform development, design, and testing of the tool [29]. This method was primarily used to ensure that development was in close collaboration with end users and to optimize acceptability, feasibility, and engagement. The EASSi tool, based on a previously published conceptual framework [30], is accessed via a tablet device and includes approximately 15 to 20 “pages” with large text on a screen. The text is intended to be viewed by both the health care professional and the patient and used as part of a shared conversation. The tool’s programming uses algorithms to provide information tailored to treatment type and partner status. An accompanying printed sign-posting sheet is also included to provide personalized support resources. The aim of this study was to assess the feasibility and acceptability of the tablet-based EASSi tool, and health care professional and patient satisfaction with the tool in routine prostate cancer care settings.

## Methods

### Study Design

A mixed-methods approach was employed according to program usage data and surveys completed by health care professionals and men with prostate cancer following use of the EASSi tool. A minimum sample size of 50 appointments was selected a priori to ensure sufficient data were gathered. Additional qualitative data exploring user perceptions were also gathered using in-depth semistructured interviews with the health care professionals and a randomly selected sample of patients. For the qualitative component, recommendations of the consolidated criteria for reporting qualitative research (COREQ) were followed [31]. Interviews were led by a researcher with extensive experience in conducting cancer research (EMcC).

### Study Population and Setting

Participants were health care professionals working in prostate cancer care and men attending routine appointments as part of treatment or follow-up. No exclusions were applied to age, treatment type, stage of the disease (for patients), or years of clinical experience (for health care professionals). Written informed consent was obtained from all participants. Ethical approval for the study was provided via the Office for Research Ethics Committees Northern Ireland (ORECNI) (reference number: 17/NI/014).

### Data Collection

**Figure 1:**
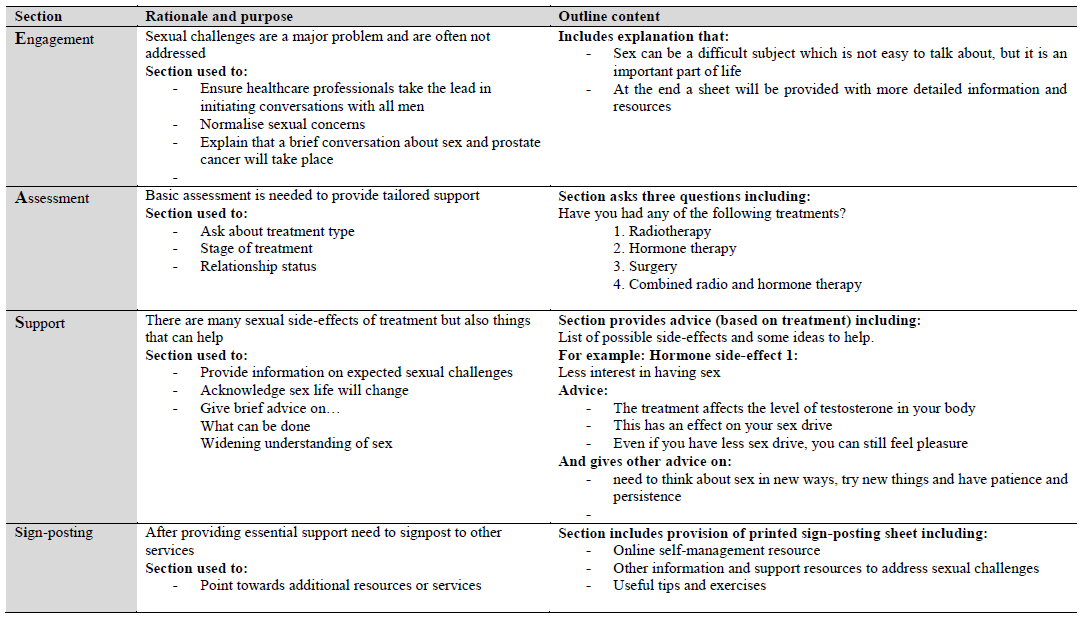
Purpose and outline content of the Engagement, Assessment, Support, and Sign-Posting (EASSi) tool.

**Figure 2:**
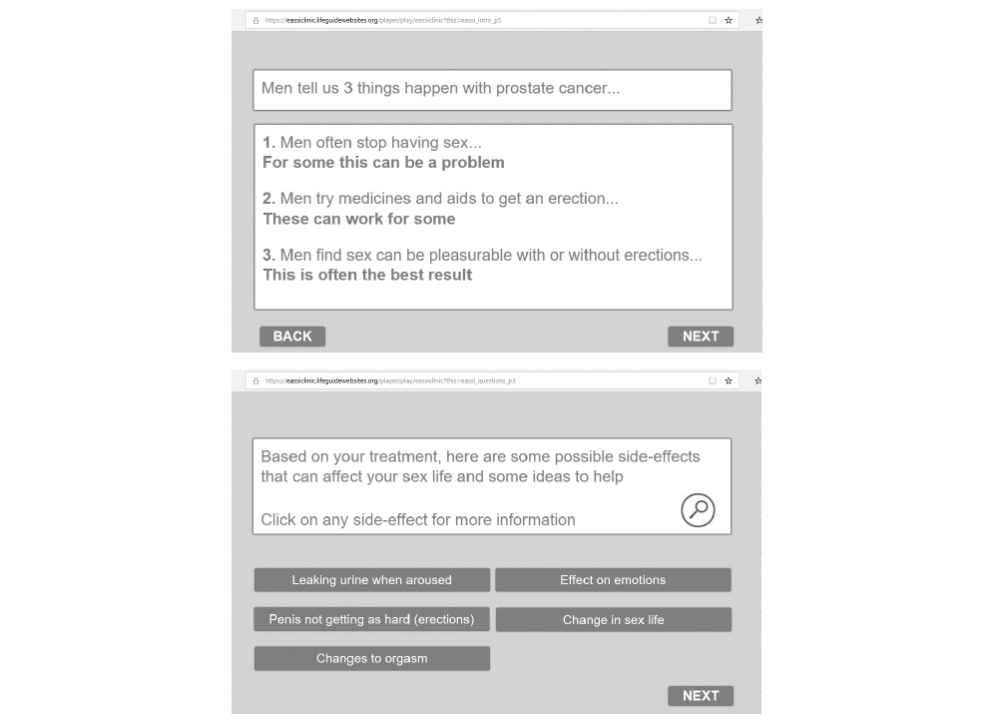
Screenshots showing pages from the "Engagement" and "Support" sections of the tablet-based Engagement, Assessment, Support, and Sign-Posting (EASSi) tool.

The EASSi tool was built using “LifeGuide” open source software [32]. Components and design features of the tool are summarized in Figure 1. Figure 2 includes screenshots of the EASSi tool. Of the four sections included, the “Engagement” section is focused on ensuring that routine sexual well-being discussions take place, acknowledging that sexual issues are not easy to discuss, and recognizing that associated side-effects of treatment can have a substantial impact. The “Assessment” section includes questions on treatment type and relationship status to provide tailored support based on responses to these “nonsensitive” questions. The “Support” section aims to provide appropriate information on common sexual challenges (relevant to treatment and relationship status). It also aims to normalize these issues and provide information on coping strategies. Lastly, the “Sign-posting” section provides details relating to other supports, including online self-management, erectile dysfunction clinic information, and resources specific to individual needs (such as information on online support groups for gay men).

All health care professionals received a standardized 30-minute familiarization and training program in use of the tool. During the evaluation, researchers working at each clinical site (CF and JC) set up the tablet (a 9-inch screen Samsung Galaxy Tab A, Android tablet) prior to each patient appointment. They then entered a unique nonidentifiable study identification and gave the tablet to the health care professional. Consecutive patients from clinic lists at four primary and secondary care sites within three National Health Service Trusts in Northern Ireland and Scotland were identified. The EASSi tool was then used as part of a discussion about sexual well-being issues following treatment. Health care professionals completed the brief survey at the end of the tool immediately after each use. Patient participants were provided with a pack containing an evaluation survey and a stamped addressed envelope for return and were asked to return the survey within 1 week of the appointment.

### Analysis

Data were gathered from program usage analytics and from postappointment surveys on usability and usefulness completed by all participants. Patients also completed a survey on sexual well-being attitudes and beliefs. Survey responses were based on four or nine-point Likert scales indicating level of agreement with each statement or question. Data were imported into SPSS Statistics for Windows version 25 (IBM Corp), which was used to perform a descriptive analysis.

Qualitative data were collected from follow-up, telephone, or face-to-face interviews conducted in quiet nonclinical rooms within a hospital setting. All interviews were conducted within 1 week of the appointment. Semistructured interview schedules were developed based on previous research [33]. These consisted of open-ended questions focused on exploring the experience of using the EASSi tool. Interviews were audio recorded and transcribed verbatim. Field notes were also recorded. These were summarized to support analysis and interpretation of data and were sent to participants for review on request. Reflexive thematic analysis was used to synthesize data [34]. Feasibility and acceptability were examined using program usage data (including duration of discussions and pages viewed), as well as responses to quantitative survey questions, which were reported as mean values and percentage agreement scores. Satisfaction with use of the EASSi tool was assessed using qualitative findings from the open-ended survey questions and from the interviews that explored participant experiences of use.

## Results

### Participant Characteristics

Eight health care professionals (three urology and oncology specialist nurses, one well-being nurse, two oncology doctors, a general practitioner, and a cancer support worker) used the EASSi tool during consecutive patient appointments. For a small number of appointments (5/94, 5%), the health care professional deemed it unsuitable to use the EASSi tool as the patient was medically unstable or was attending the appointment with a family member (other than a partner). The EASSi tool was therefore used during 89 patient appointments. Of these, 53 were at clinical sites in Northern Ireland (primary care: n=4; secondary care: n=49) and 36 were at sites in Scotland (secondary care: n=26; posttreatment well-being clinics: n=10). Twenty-six patients (29%) had surgical treatment only, with the majority having had surgery within the past 6 months (n=22, 85%). Seven patients (8%) had or were receiving radiotherapy, while 9 (10%) were on ongoing hormone therapy only. The remainder (n=47, 53%) had or were receiving combined radiotherapy and hormone therapy. Most patients reported having had no previous sexual care discussions with a health care professional (n=52, 58%). The majority had a partner (n=83, 93%).

### Program Usage Data

The mean duration of conversations that took place using the EASSi tool was 6.01 minutes (SD 2.91), ranging from 2.62 to 11.74 minutes. The greatest amount of time was spent in the “Support” section (3.32 minutes, SD 1.12), with 1.03 minutes (SD 0.74) spent in the “Engagement” section, 0.59 minutes (SD 0.33) spent in the “Assessment” section, and 1.23 minutes (SD 0.74) spent in the “Sign-posting” section. Approximately two side-effect pages were viewed during each use; however, this number ranged from 0 to 6. The most frequently viewed side-effect pages were on “loss of erections” and “loss of interest in sex.” No technical issues with use of the tablet were identified during use.

### Postappointment Survey Findings

Surveys completed after use (n=89 appointments) indicated that health care professionals viewed the EASSi tool as being valuable for helping to talk about sexual well-being (mean score 7.7/9, SD 1.3; 91% agreement) and for providing relevant information to the patient (mean score 7.1/9, SD 1.5; 92% agreement). The tool was also viewed as simple to use (mean score 8.3/9, SD 0.9; 98% agreement). Thirty patients did not return their postal surveys, and evaluation data were therefore available for 59 (66%) of the 89 patients who took part in a sexual well-being discussion using the EASSi tool. Patient surveys also indicated that the tool was seen as helping the sexual well-being discussion (mean score 3.4/4, SD: 0.8; 85% agreement) and providing relevant information (mean score 3.3/4, SD 0.7; 85% agreement). While free text comments made by health care professionals and patients in the survey also indicated that the EASSi tool was seen as useful, there were differing perspectives. For example, after some appointments, health care professionals reported that the tool was less useful as the patient was “not concerned” about sexual issues, whereas patients (commenting on the same appointment) were typically more positive, stating how valuable the conversation was (Table 1). This was further supported by other data from the surveys, which indicated that patients agreed with the statement that talking about sexual well-being was important to them (mean score 3.5/4, SD 0.5; 88% agreement). The additional survey questions around sexual attitudes and beliefs identified that patients disagreed with the statement that they were uncomfortable discussing sexual well-being during appointments (mean score 1.8/4, SD 1.4; 46% agreement) (Table 2).

**Table 1:** Examples from individual appointments demonstrating where the perspectives of health care professionals and patients on “usefulness” of the EASSi tool differed or were consistent.

Health care professional views on “usefulness” of the discussion	Patient views on the same discussion	Views differed (−) or were consistent (+)
“...patient and his wife expressed they were not concerned about absent sexual function” [Clinical nurse specialist, Uro-oncology]	“I read through the information on the tablet and found it informative” [6 months after radiotherapy, ongoing hormone therapy, has a current partner]	−
“...patient was keen to focus on fatigue and emotions rather than sexual function” [Clinical nurse specialist, Surgical oncology]	“it was useful finding out about side-effects on your sex life in general, including the information on erectile dysfunction” [less than 6 months after radiotherapy, ongoing hormone therapy, no current partner]	−
“...patient was not sexually active and not really concerned about sex life at all” [Clinical nurse specialist, Uro-oncology]	“dealing with the nurse about sex was far more informative and helpful than dealing with the doctor. I could have done with this type of appointment when first diagnosed” [more than 6 months after radiotherapy, ongoing hormone therapy, has a current partner]	−
“...they were not concerned. They were able to get erections, with dry orgasms” [Clinical nurse specialist, Urology]	“...it made the discussion easier, especially around lack of sex drive and the problems resulting from treatment. The conversation could have actually been longer” [more than 6 months after radiotherapy, ongoing hormone therapy, has a current partner]	−
“...it was very useful, it made discussing the topic easier and covered more depth and detail. Very easy to discuss delicate area” [General practitioner]	“...it helped with understanding the positives of aftercare after prostate cancer and with knowing there is good support after surgery. The info provided was helpful” [more than 6 months after surgery, has a current partner]	+
“...it prompted me to suggest getting more advice from the GP and ask about a trial of a PDE5 inhibitor” [Clinical nurse specialist, Uro-oncology]	“...getting the tablet explained was good, it helped a lot” [less than 6 months after radiotherapy, has a current partner]	+
“...This gentleman was very open to the discussion and use of the technology to assist the conversation. Made conversation easier. He recognized himself in the issues presented” [Nurse, Oncology]	“...having read all the literature given to me at the start (several times) I knew what to expect but it is helpful to discuss where you are and to set yourself some goals” [less than 6 months after radiotherapy, has a current partner]	+

**Table 2:** Mean scores and percentage agreement for statements exploring patient sexual attitudes and beliefs.

Question	Score (/4)^a^, mean (SD)	Percentage agreement
I understand how my treatment for prostate cancer might affect my sexual well-being	3.5 (1.1)	89
I am uncomfortable talking about sexual issues with health care professionals	1.8 (1.4)^b^	46
Health care professionals should make time to discuss sexual well-being with me	3.2 (1.2)	80
I feel confident that health care professionals have the ability to address my sexual concerns	3.4 (1.1)	85
Discussing sexual well-being is essential to my health outcomes	3.1 (1.3)	78
Some health care professionals are more comfortable talking about sexual issues with me than others	2.1 (1.2)	53
I expect health care professionals to ask me about my sexual concerns	3.2 (1.3)	80

^a^Score of 1, strongly disagree; 2, disagree; 3, agree; 4, strongly agree.

^b^Indicates disagreement with the statement.

### Qualitative Interview Findings

Semistructured interviews were held with all eight health care professionals who used the tool and with a randomly selected sample of men (n=10). Interviews lasted approximately 1 hour. The analysis identified three key themes around use of the EASSi tool.

#### Theme 1: Moving From Optional to Routine Conversations

##### Health Care Professionals

Health care professionals acknowledged that using the EASSi tool increased the frequency with which they discussed sexual well-being and that it had an immediate positive impact by enabling easier initiation of discussions with a wider group of patients, including those they might not have conversations with if not using the tool. They also observed that conversations were associated with less awkwardness than they had expected. While some felt there were still men for whom it would be inappropriate to discuss sexual well-being, it was reflected upon by others that this represented a degree of “gate-keeping,” which could be used as a mechanism to avoid initiating conversations. Health care professionals found that the purposeful design of the tool helped to “manage” the conversation and provided a mechanism to direct the conversation, ensuring greater consistency and leading to a less “ad-hoc” approach when discussing sexual concerns with patients.

##### Patients

Patients welcomed the discussion, stating how it was presented in a comfortable and professional manner. Patients also recognized how the role of the partner was acknowledged using the tool. They also stated that the tablet format was straightforward, and they valued the limited words on the screen. One patient made the following statement:

Actually, it was very easy to follow, just a few words on each screen… we could stop and discuss anything at any time point.

Patient #7, male

#### Theme 2: Improving Depth of Conversations and Support Provided

##### Health Care Professionals

Health care professionals found that the tool enhanced conversations and facilitated a “higher level” of patient involvement. It was acknowledged that before using the EASSi tool, sexual issues were often not discussed during appointments or were only addressed superficially by providing limited information on erectile dysfunction. Health care professionals described how a greater “depth” of information was provided, including simple but clear information on how patients’ sexual lives could be impacted and practical advice on how to manage these issues. Expectations around recovery were addressed and a wider understanding of intimacy was introduced, moving away from a focus on erectile dysfunction only. One professional commented as follows:

…without using [it] today the value of the consultation would have been hugely inferior.

Consultant urologist, male

Some health care professionals described how discussions were “collaborative” and provided more than just delivery of information. The pages outlining treatment side-effects were seen as being the most interactive element, introducing an opportunity for patients to “take the lead” in identifying side-effects of interest to them. Following the first use, health care professionals reported becoming more confident using the tool, integrating it into practice, sharing the screen with patients, and adapting the content to suit their own communication style. There were practical issues reported. For example, some men did not have their glasses with them or were reluctant to read the screen. Such issues were often compensated for by the health care professional taking a greater lead in the discussion.

The “Sign-posting” pages and accompanying printed hand-out were regarded as important components by health care professionals. Their value was seen in terms of the ability to direct patients toward resources appropriate to their needs and advice to “get started.” They were also seen as a useful “prompt or reminder,” reinforcing key messages from the discussion.

##### Patients

Patients reported that conversations were useful and straightforward. For some, it was the first meaningful discussion about the sexual consequences of treatment. One patient commented as follows:

Apart from before treatment when I was told that my erections would go, nobody has mentioned the sex thing. After chatting to the nurse last Friday using the computer, I was able to better understand why I was feeling so different.

Patient #9, male

Some reported that the tool provided a “sense of control” by selecting information that was most relevant to them. One patient commented as follows:

I could press what buttons I wanted…I never would have asked out loud about dry orgasms!

Patient #2, male

Others indicated that they felt comfortable just listening to the health care professional. One patient commented as follows:

Sex is not something that bothers me at the moment but I’m glad it was mentioned, and I think it should be talked about.

Patient #6, male

#### Theme 3: Normalizing Sexual Well-Being Issues in Routine Practice

##### Health Care Professionals

Health care professionals described how the EASSi tool and discussing sexual well-being routinely had alerted them to how important sexual well-being care is. They described how discussions being a standard aspect of care might result in men being more comfortable with initiating future discussions. Examples of this included patients being more able to seek out further information (from the sign-posting sheet) or discuss issues with other health care professionals, even after active treatment. One professional made the following comment:

It might not be right now, but they now know that they can talk about it with you.

Specialist oncology nurse, female

For more experienced clinicians, the EASSi tool was regarded as a way of embedding sexual well-being conversations into routine practice. Having used the tool with several patients, one professional made the following statement:

Providing information about sexual care simply needs to be something that everyone in the clinic just knows and that we do it as routine.

Consultant urologist, male

##### Patients

Overall, patients felt that the tool helped “normalize” sexual issues, treating the topic in the same way as other symptoms. They also felt reassured that their experiences were not unique and were more common than they previously thought.

## Discussion

### Principal Findings

This study evaluated a systematically developed tool designed to facilitate and structure sexual well-being discussions in prostate cancer care. The tablet-based EASSi tool was used as part of sexual well-being conversations in primary and secondary care settings. Overall, health care professionals and patients found the tool to be acceptable and appropriate and were satisfied with its use during appointments. It was found to facilitate brief but meaningful discussions that were feasible as part of routine appointments by providing a “standardized” mechanism to initiate discussions, ensuring that sexual well-being was consistently raised as a topic. It was also reported that the tool was useful for improving overall communication around sexual well-being through provision of fundamental information and support tailored to treatment and relationship status. Health care professionals and patients did have contrasting perspectives around the need for use of the tool. There was evidence that some health care professionals may have underestimated and downplayed the value of the sexual well-being discussions to patients, who regarded the discussions as valuable and important. Patients also highlighted some regret that they had not had similar discussions prior to or earlier in treatment. While there are valid clinical reasons why a sexual well-being discussion might not take place during an appointment, for example, high levels of patient distress and medical instability, “gate-keeping” or assumptions about readiness or willingness to discuss sexual issues can lead to patients not receiving appropriate information and support [35]. Ensuring that discussions occur routinely should be an important part of supporting patients to manage alterations to sexual function and expectations around recovery [16,36].

### Strengths and Limitations

The particular strengths of the EASSi tool were that it was concise and simple to use, included an engagement section to initiate conversations in a standard manner that limited potential embarrassment, used “nonsensitive” language throughout, and provided support based on individual need. Onward referral to other more specialist services included within the “Sign-posting” section alongside other readily accessible support options was also seen as valuable. Another perceived strength of the tool was its flexibility, with scope to facilitate a brief conversation or be used as a part of a more involved discussion. A limitation of the study was that the perspectives of the 30 (34%) patients who did not return the evaluation survey after the appointment were unknown.

### Study Implications

This evaluation provides initial support for use of the EASSi tool in practice. Findings indicated that the tool was appropriate and acceptable for use and promoted delivery of routine sexual care for men with prostate cancer. The EASSi tool incorporates components aimed at ensuring that discussions are more routine and that essential support is provided as part of prostate cancer care. These techniques include changes to the physical environment (the tablet device itself), as well as delivery of appropriate information and the use of patient prompts in the form of a printed handout used to reinforce key messages and point to effective evidence-based self-management resources. The theoretical underpinning of the EASSi tool may be similar to models, such as the 5 A’s approach (ask, assess, advise, agree, and assist), which have been used as frameworks to initiate, standardize, and guide brief behavior change interventions [37]. The tool can be used across settings and without specific training or expertise in sexual care counselling. In addition, the tool might be used during pretreatment consultations to assist with improving a patient’s awareness of the possible impact of different treatment options on sexual well-being and to reduce decisional regret, which is often experienced when patients feel they had a passive role in treatment decision-making [9,38]. The tool could also be viewed by patients alone (not only during appointments with a health professional) to help provide information on the side-effects of treatment and on approaches to help manage these effects. One other potential application that could be explored further is use of the tool to structure sexual well-being conversations during remote appointments delivered via telephone or videoconferencing facilities [39].

The tool was identified as being useful for addressing barriers to sexual well-being discussions and supporting health care professionals to initiate discussions by facilitating brief discussions that normalized sexual issues and provided patients with essential support. The findings do suggest that health care professionals may underestimate how important sexual well-being discussions are for patients. Additional research should be conducted to help health care professionals explore their views on sexual issues and overcome barriers to discussing sexual well-being with patients. Further work will also be conducted to evaluate the effectiveness of using the tool in different cancer care settings.

### Conclusions

The EASSi tool may provide a practical format to guide routine sexual well-being discussions in clinical practice. The tool also includes tangible take home messages for prostate cancer survivors in the form of a printed “sign-posting” sheet. Use of the tool in practice may promote increased engagement around sexual well-being to ensure fundamental support is provided to men and their partners. This could potentially address current gaps in the lack of routine provision of sexual well-being support for men living with prostate cancer.

## Abbreviations

EASSi: Engagement, Assessment, Support, and Sign-posting
